# Social protection as a right of people affected by tuberculosis: a scoping review and conceptual framework

**DOI:** 10.1186/s40249-023-01157-1

**Published:** 2023-11-22

**Authors:** Melisane Regina Lima Ferreira, Rafaele Oliveira Bonfim, Pedro Augusto Bossonario, Venisse Paschoalin Maurin, Ana Beatriz Marques Valença, Paula Daniella de Abreu, Rubia Laine de Paula Andrade, Inês Fronteira, Aline Aparecida Monroe

**Affiliations:** 1 University of Sao Paulo at Ribeirao Preto College of Nursing, Ribeirao Preto, Sao Paulo, Brazil; 2 Global Health and Tropical Medicine, Institute of Hygiene and Tropical Medicine, NOVA University Lisbon, Lisbon, Portugal; 3 National School of Public Health, Public Health Research Center, Comprehensive Health Research Center, NOVA University Lisbon, Lisbon, Portugal

**Keywords:** Tuberculosis, Social welfare, Public policy, Human rights, Government program, Review

## Abstract

**Background:**

Tuberculosis is an infectious disease strongly influenced by social determinants closely associated with cycles of poverty and social exclusion. Within this context, providing social protection for people affected by the disease constitutes a powerful instrument for reducing inequalities and enhancing inclusion and social justice. This study aimed to identify and synthesize strategies and measures aimed at ensuring social protection as a right of people affected by tuberculosis.

**Methods:**

This is a scoping review, with searches conducted in six databases in February 2023. We included publications from 2015 onwards that elucidate strategies and measures of social protection aimed at safeguarding the rights to health, nutrition, employment, income, housing, social assistance, and social security for people affected by tuberculosis. These strategies could be implemented through policies, programs, and/or governmental agreements in any given context. The data extracted from the articles underwent descriptive analysis and a narrative synthesis of findings based on the dimensions of social protection. Additionally, we developed a conceptual framework illustrating the organizational and operational aspects of measures and strategies related to each dimension of social protection identified in this review.

**Results:**

A total of 9317 publications were retrieved from the databases, of which sixty-three publications were included. The study’s results highlighted measures and strategies concerning the social protection of people affected by tuberculosis. These measures and strategies revolved around the rights to proper nutrition and nourishment, income, housing, and health insurance, as well as expanded rights encompassing social assistance and social welfare. It was reported that ensuring these rights contributes to improving nutritional status and the quality of life for individuals with tuberculosis, along with reducing catastrophic costs, expanding access to healthcare interventions and services, and fostering TB treatment adherence, thereby leading to higher rates of TB cure.

**Conclusions:**

Our findings identify social protection measures as a right for people affected by tuberculosis and have the potential to guide the development of evidence-based social and health policies through collaboration between tuberculosis control programs and governmental entities.

**Supplementary Information:**

The online version contains supplementary material available at 10.1186/s40249-023-01157-1.

## Background

Tuberculosis (TB) is an infectious disease highly influenced by social determinants, maintaining a direct relationship with cycles of poverty and social exclusion. This leads to an increased risk of illness among socially neglected populations, exacerbating social inequalities and health inequities [1]. Health is a focal point across all 17 Sustainable Development Goals (SDGs), notably within the third goal: Health and Well-being. For TB, objective 3.3 aims to end the disease epidemic, alongside other communicable diseases, by 2030. It is crucial to recognize that this objective is intrinsically intertwined with goals related to reducing extreme poverty and inequalities [2].

Therefore, it is essential to reflect upon the interconnected challenges to attain these objectives. This is why the World Health Organization (WHO) launched The End TB strategy. This strategy aims to eliminate the disease as an endemic by reducing mortality rates by 90% and incidence rates by 80% by 2030, ultimately eliminating it by 2050. Another goal is to ensure that any person with TB does not have to bear catastrophic costs due to the disease [2, 3].

The right to health is one of several rights intertwined in the TB response, necessitating coordination with a spectrum of underlying rights including access to clean water, nourishment, proper nutrition, housing, healthy occupational and environmental conditions, education, and income [7]. Thus, addressing the implications of health vulnerabilities requires an expanded human rights perspective to effectively combat TB, which includes social protection as a right within the human right to health.

A fundamental pillar for ending TB is built upon robust policies and support systems aimed at the social protection of people affected by the disease. This involves reducing poverty and intervening in other determining factors associated with illness by TB, such as food scarcity, malnutrition, decreased income, poor living conditions, and lack of social support, such as emotional, instrumental, informative, community support, among others [3, 4]. Hence, it is urgent to implement rights-ensuring policies and strengthen healthcare systems, particularly focusing on vulnerable social populations [1].

Social protection involves policies and programs designed to guarantee human rights, prevent risks and vulnerabilities throughout life, mitigate damages and negative impacts, and provide support to meet basic population needs. This protection encompasses various areas of the human rights, including health, social security, social assistance, employment security, and other aspects related to economic and social well-being [5]. Social protection policies and programs are recognized as a powerful tool for poverty reduction, promoting equity, social justice, inclusion, and social solidarity [5, 6].

Previous systematic reviews have examined the influence of socioeconomic interventions on the social determinants of TB and the effects of social protection measures on TB outcomes, including heightened cure rates, decreased lost to follow-up cases, and reductions in poverty-related issues like food insecurity and malnutrition [8–12]. However, none of these reviews addressed these aspects from the perspective of the rights of people with TB. These questions emphasize the need for resources concerning this matter to better inform decision-making based on knowledge synthesis materials.

We decided to conduct a scoping review given the breadth and coverage of existing evidence within a specific field of knowledge, incorporating concepts, examples, and identifying knowledge gaps [13]. Therefore, the objective of this scoping review was to identify and synthesize strategies and measures aimed at ensuring social protection as a right of people affected by TB.

## Methods

This scoping review followed the methodology developed by the Joanna Briggs Institute Reviewer’s Manual for Scoping Reviews [14], as well as incorporating recommendations from the Preferred Reporting Items for Systematic Reviews and Meta-Analyses—Extension for Scoping Reviews (PRISMA-ScR) [15]. The study was developed through a series of steps: (1) formulation of the guiding question; (2) identification of relevant publications; (3) selection of pertinent publications; (4) data extraction; (5) data analysis; and (6) synthesis of scientific evidence. This study was registered on the Open Science Framework Registries (OSFREGISTRIES) platform (https://doi.org/10.17605/OSF.IO/WX3KM).

### Formulation of the guiding question

The research question, “What is the scientific evidence regarding strategies and measures aimed at social protection as a right of people affected by tuberculosis in the international context?” was formulated following the criteria of the PCC strategy [15]. In this strategy, the acronym P (population) corresponds to people affected by tuberculosis; C (concept) represents strategies or measures focusing on social protection; and C (context) refers to the global setting.

We used this strategy to understand the scope of the research topic and its relationship with the population, concept, and context of interest, without limiting ourselves to specific interventions.

### Identification of relevant publications

We began this process by identifying controlled vocabulary from the PCC strategy keywords, which were indexed through the Health Sciences Descriptors (*Descritores em Ciências da Saúde—DeCS*) in English, Portuguese, and Spanish. For English keywords, we consulted the Medical Subject Headings (MeSH).

The search terms implemented during this step encompassed ‘TB’ and the diverse dimensions of social protection, including terms like ‘public policy’, ‘social welfare’, ‘public assistance’, ‘health policy’, ‘income’, ‘financial support’, ‘work’, ‘food assistance’, ‘food security’, ‘food insecurity’, ‘social security’, ‘government financing’, ‘government program’, ‘social work’, ‘housing’, ‘human rights’, and ‘socioeconomic rights’. These terms encompass the controlled vocabulary used for searches, which was supplemented by free vocabulary (synonyms) used in the writing of publications and found in previous searches conducted in the selected databases for the study. See the search strategies in Additional file 1.

The searches were carried out by a researcher (MRLF) in February 2023, utilizing the identified vocabulary in conjunction with the boolean operators “AND” and “OR”. The search strategies were customized for each database: Scopus, Web of Science, Medical Literature Analysis and Retrieval System Online (MEDLINE), Latin American and Caribbean Health Sciences Literature (LILACS), Embase^®^, and Cumulative Index to Nursing and Allied Health Literature (CINAHL).

It is important to highlight that our LILACS searches involved both controlled and free vocabulary in Portuguese, English, and Spanish. Conversely, only English vocabulary was employed for searches within the other databases.

### Selection of pertinent publications

After completing database searches, all identified data were exported to the Rayyan application (Qatar Computing Research Institute’s Rayyan application, Doha, Qatar) [16] to facilitate the elimination of duplicated articles and the selection of pertinent publications. To accomplish this, two independent researchers were blinded to each other's screening and examined the titles and abstracts of the publications (MRLF and ROB). In cases of disagreements, a third reviewer (RLPA) was consulted.

Publications with potential eligibility underwent a secondary selection process involving a comprehensive reading of their full content. In cases where the complete text was inaccessible online, attempts were made to contact the authors of the selected papers. Should these attempts prove unsuccessful, the respective article was excluded from the study.

Considering that social protection of people affected by TB encompasses multiple dimensions of poverty and directly pertains to the social determinants of health, our focus was on identifying publications that present strategies and measures aimed at safeguarding rights related to health, nutrition, employment, income, housing, social assistance, and social security. These strategies and measures could be implemented through policies, programs, and/or governmental agreements in any given context.

Our inclusion criteria encompassed peer-reviewed journal publications from 2015 to 2023. We chose 2015 as the starting point since it marked the year when the WHO published The End TB strategy, with the goal of ending the disease as an endemic condition by 2030 and eliminating it altogether by 2050. This publication introduced the concept of social protection to people affected by TB as a right and a cornerstone of the strategy to combat TB, relying on robust policies and support systems [2].

Furthermore, we included studies in English, Portuguese, and Spanish, covering both primary and secondary research that addressed any form of TB (pulmonary, extra-pulmonary, and drug-resistant) using diverse methodologies (quantitative, qualitative, or mixed methods) and methodological designs. We excluded protocols, manuals, guidelines, technical notes, content from publicly available websites, and studies that only described direct and/or indirect socioeconomic effects or consequences of TB treatment without detailing corresponding measures and strategies for mitigation.

Studies in this review were presented in a flowchart, as recommended by the PRISMA-ScR [15]. Reasons for exclusions were documented for all full-reading articles.

### Data extraction

The extraction of crucial information was conducted by the authors (ROB, PAB, ABMV, PDA, and VPM), typed into Microsoft Excel (Microsoft Corporation, Redmond, USA), and subsequently reviewed by a second reviewer (MRLF). The extracted details encompassed the title, authors, language, year of publication, study country, country categorization (high or low TB incidence), journal of publication, objective, methodological design, publication type, study population or sample, TB type, special population, dimensions of social protection, primary strategy or measure for social protection, name of the strategy or measure, funding source for the strategy or measure, objective of the principal strategy or measure, operational mechanism of the strategy or measure, conditionalities associated with the strategy or measure, co-implemented strategies or measures, and key results.

Furthermore, we extracted “TB-specific” or “TB-sensitive” information related to social protection measures or strategies, aiming to comprehend which proved most effective or provided superior results in conjunction with TB treatment and management. TB-Specific measures benefit individuals affected by the disease and their families, and are integrated into existing TB treatment programs. On the other hand, TB-sensitive measures are part of a broader framework of social protection aimed at the general population, potentially influencing TB outcomes by bolstering economic resilience and alleviating poverty [17].

### Data analysis and synthesis of scientific evidence

After the extraction process, we carried out a descriptive analysis of the overall characteristics of the studies included and conducted a narrative synthesis of the results based on the dimensions of social protection. It should be noted that this scoping review did not include a formal consultation exercise.

Upon synthesizing the scientific evidence from this scoping review, we summarized and categorized the findings, elaborating a conceptual framework that represents the organizational and operational elements of measures and strategies pertaining to each identified dimension of social protection. Furthermore, aspects associated with the planning and implementation of policies, programs, and/or governmental agreements on the international stage, encompassing social protection as a right of people affected by TB, were delineated based on three interrelated components: “structure”, which referred to social protection measures and strategies as well as other resources or policies concurrent with their provision; “process”, which involved the planning, financing, and execution of social protection measures and strategies; and “results”, which represented the effects of social protection measures and strategies in addressing the disease or TB treatment outcomes.

This study rigorously followed ethical aspects related to transparency in data extraction to ensure the inclusion of studies with both methodological and ethical foundations. In addition, we used a non-stigmatizing language related to TB [18].

### Quality assessment of included literature

In accordance with the guidelines outlined in PRISMA-ScR [15], we did not perform a quality assessment of the included studies.

## Results

**Fig. 1:**
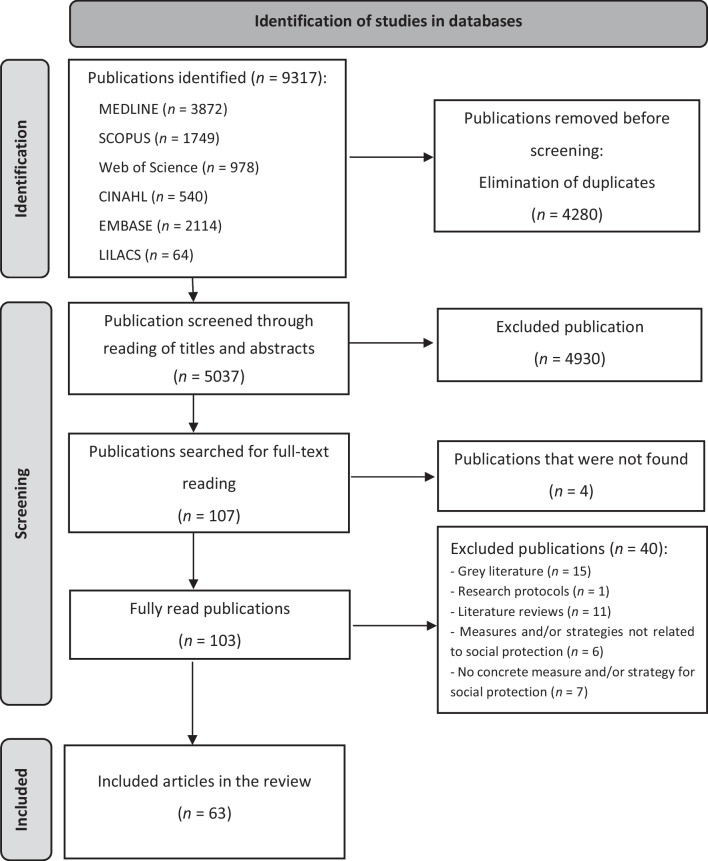
PRISMA flowchart of article searches and publications included in this scoping review. Adapted from [19]

A total of 9317 publications were identified in the databases. Among them, 4280 were excluded due to duplication, and 4945 were eliminated after reviewing titles and abstracts. Out of the remaining 107 publications subjected to full-text review, four were excluded because their complete content could not be located. Consequently, 103 publications were considered eligible for full-text reading, and of these, 63 were included in the review (Fig. 1).

### General characteristics of included studies

**Fig. 2:**
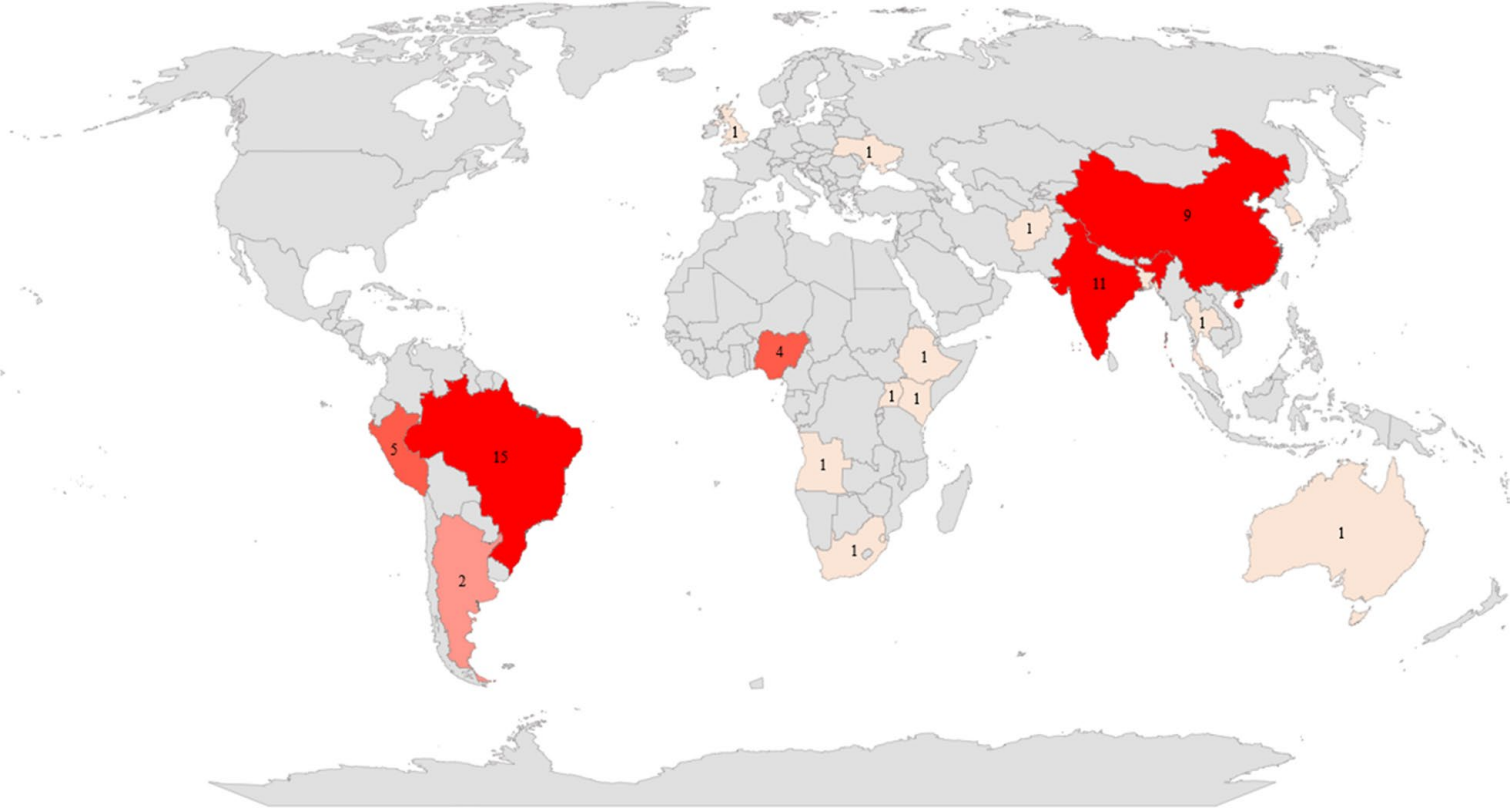
Countries* of the studies included in this scoping review. * Afghanistan (*n* = 1), South Africa (*n* = 1), Angola (*n* = 1), Argentina (*n* = 2), Australia (*n* = 1), Bangladesh (*n* = 1), Brazil (*n* = 15), China (*n* = 9), Singapore (*n* = 1), Republic of Korea (*n* = 1), Eswatini (*n* = 1), Ethiopia (*n* = 1), India (*n* = 11), Nigeria (*n* = 4), Peru (*n* = 5), Kenya (*n* = 1), United Kingdom (*n* = 1), Thailand (*n* = 1), Ukraine (*n* = 1), Uganda (*n* = 1), and group of countries (*n* = 3)

Regarding the general characteristics of the included studies, five were published in 2015, 10 in 2016, eight in 2017, 13 in 2018, 11 in 2019, five in 2020, four in 2021, six in 2022, and one in 2023. Predominantly, the study locations were countries with a high TB incidence (*n* = 52), and a larger number of them were conducted in Brazil (*n* = 15), India (*n* = 11), China (*n* = 9), Peru (*n* = 5), and Nigeria (*n* = 4) (Fig. 2). Three studies encompassed a group of countries, providing a more global analysis of the implemented measures and strategies for social protection.

Regarding the study population, people affected by TB, healthcare professionals and managers, intradomestic contacts, community members, as well as various population groups in social vulnerability, such as migrants, refugees, homeless people, incarcerated people, indigenous people, *quilombolas* [descendants of Africans who were enslaved and who fled from slavery in Brazil during the colonial centuries and formed independent communities known as *quilombos*], and populations residing in rural and shantytown areas, were included. The publications encompassed all forms of TB, including sensitive forms of pulmonary TB, extrapulmonary TB, and mixed TB, as well as drug-resistant TB (DR-TB) and multidrug-resistant TB (MDR-TB).

More than half of the included studies presented specific measures and strategies focused on people affected by TB, another 16 articles explored TB-sensitive measures and strategies, and three studies listed the provision of both (TB-specific and TB-sensitive) for promoting social protection for individuals affected by the disease.

### Objectives and methods of included studies

Regarding the objectives of the studies, the majority of publications were designed to analyze the influence of measures and strategies aimed at social protection on the diagnosis of TB, incidence, adherence, and, primarily, on the outcomes of treatment for both drug-sensitive TB and drug-resistant cases. Other publications focused on the impact of social protection measures on addressing risk factors for TB illness, alleviating catastrophic costs, ensuring food security, malnutrition and impoverishment during treatment, while some studies solely aimed to explore the quantity or proportion of people affected by TB who benefited from some form of social protection-oriented measure or strategy.

As for organizational aspects, some authors aimed to identify the coordination of healthcare, equity, and access to social protection, as well as the experiences, barriers, and facilitators of the implementation process of these measures or strategies.

There was a predominance of quantitative studies, including 17 cohort studies, five descriptive studies, five ecological studies, four randomized clinical trials, three intervention studies, two cross-sectional studies, one quasi-experimental study, one case–control study, and one evaluative study. In the seven qualitative studies, authors utilized phenomenological approaches, participatory action research, and grounded theory to discuss their findings. Additionally, 11 publications were included using a mixed-methods approach, employing various types of studies.

### Conceptual framework

The measures and strategies identified in the included studies were organized into four dimensions related to social protection as a right of people affected by TB: the right to proper nutrition and nourishment, income, housing, and health insurance. A fifth dimension was created to describe expanded rights that were concurrently offered and included, in addition to the rights already mentioned, the right to social assistance, social security, and the right to transportation.

**Fig. 3:**
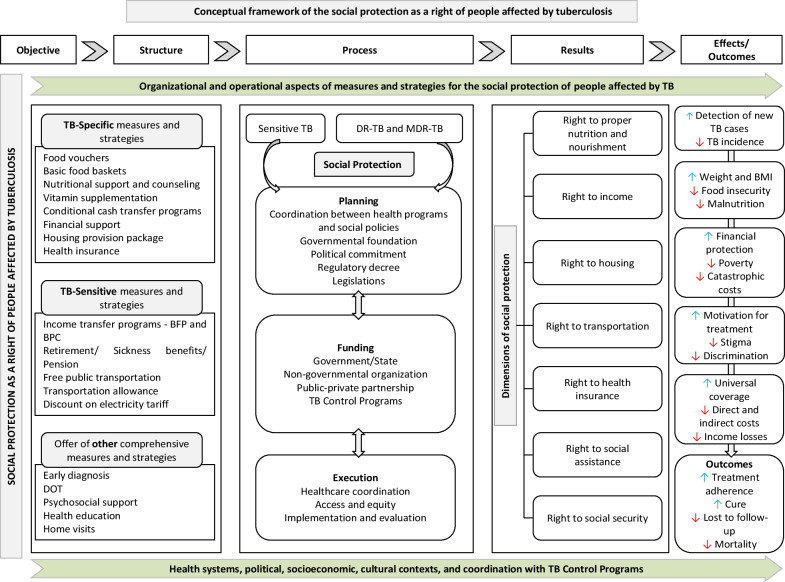
Conceptual framework of the social protection as a right of people affected by tuberculosis. *BFP* Bolsa Família Program; *BMI* Body Mass Index; *BPC Benefício de Prestação Continuada* (Continuous Cash Benefit); *DOT* Directly Observed Treatment; *DR-TB* Drug-resistant tuberculosis; *MDR-TB* Multidrug-resistant tuberculosis; *TB* Tuberculosis

It is important to note that none of the studies included addressed the right to work; therefore, this dimension remained unexplored within the scope of this review. The dimensions were summarized in Table 1 and Fig. 3, outlining the organizational and operational aspects as well as the planning, execution, and potential effects on TB treatment and management.

**Table 1:** Summary of measures and strategies identified in the included studies (*n* = 63)

Dimensions of social protection	Strategy or measure	Organizational and operational aspects		
		Objective of strategies or measures	Conditionalities	Results and/or effects
Proper nutrition and nourishment	Monthly food stipend [27, 29]	- Increase adherence to TB treatment and DOT [20, 24, 29] - Reduce the severity of the disease and enhance the quality of life for people with TB [21] - Enhance TB treatment outcomes [20, 21, 25–27] and the nutritional status of people affected by TB [28, 30] - Alleviate the indirect costs of TB treatment [23] and the heightened vulnerability to food insecurity among families impacted by TB [24]	- Individuals with TB aged 18 years or older [21, 25, 29] or children aged 2 to 14 years [25] - Individuals with TB living below the poverty line [26], with low income, who have adhered to DOT [27] or conventional TB treatment [24] - Australian Aboriginal population [28] - Individuals with TB with some degree of malnutrition [23] - Individuals with TB who have a bank account [22]	Improved TB treatment outcomes and higher treatment completion rates [27, 29]
	Monetary values for purchasing basic food baskets [20, 22, 25] Provision of food products [24] Weekly food baskets [28]			Increased rates of therapeutic treatment success and treatment follow-up losses [20] Significant weight and BMI gain among individuals with active TB [25]
	Nutritional support [21–23, 26, 30]			Enhancement of nutritional status and quality of life for individuals affected by TB [21] Decrease in treatment failure [26]
	Nutritional counseling, vitamin supplementation (vitamin A and B6), and fortified/therapeutic foods [23]			Reduction in the lost to follow-up [23]
Income	Specific Income Transfer Program for TB [31–40]	- Reduce the financial difficulties faced by people affected by TB and their family members [41, 53] - Achieve better success rates in TB treatment [32–34, 36, 38, 39, 53] and MDR-TB [40] - Increase adherence to TB treatment [31, 36, 51, 52] - Mitigate the direct and indirect catastrophic costs of TB treatment [31, 39, 52, 55]	- Individuals with drug-susceptible TB [31–39, 51, 52] - Individuals affected by antimicrobial-resistant TB [35, 39, 40, 53] - Family members of individuals with TB [54] and households with people with TB in situations of poverty [45, 55] - People affected by TB with a bank account [33]	Increase in the level of acceptance, motivation, and adherence to treatment [31] Improvement in treatment success for individuals with MDR-TB [40]
	Regulatory Decree 170/91 of Law 10.436 [34, 35]			Higher success rates in TB treatment and a lower rate of loss to follow-up in the group enrolled in the program, compared to the non-enrolled group [34] Better treatment progress in cases of individuals with MDR-TB [35]
	CRESIPT [36–39]			Families of people affected by TB are less likely to incur catastrophic costs [37, 39] Success in TB treatment [38]
	Sensitive Income Transfer Program for TB: - BFP [41–50] - BFP and BPC [42, 43]			Better treatment outcomes in the group that was part of the BFP [41, 44, 50] In Brazilian municipalities with high BFP coverage, the TB incidence rate [46] and TB mortality rate [45] were significantly reduced compared to those with low and medium coverage Among the indigenous population, it was identified that the BFP had a protective effect against active TB [48]
	Specific financial support [51–56]			Better treatment outcomes among individuals with MDR-TB [51–54] Reduction in loss to follow-up [52]
Housing	Housing Provision Package [57]	Improving TB treatment outcomes among homeless individuals [57]	Homeless population with TB recruited through hospital outreach [57]	High treatment success rate [57]
Health insurance	Reimbursement for individuals with TB [58–61, 63]	- Reduce the catastrophic costs incurred by people with MDR-TB [64] - Alleviate the financial burden among individuals affected by TB during treatment [58, 60, 62, 63] - Provide health protection against catastrophic diseases, such as TB [59] - Achieve equity in the utilization of health insurance schemes among both poor and non-poor people with TB [65]	- People with MDR-TB in the public and private sectors linked to the RNTCP [64, 65] - Individuals with TB and health insurance coverage [58] - People with drug-sensitive TB [63] and low income [66]	Sharp decline in the severity of catastrophic costs during TB treatment [58, 60] Effective improvement in access to and utilization of inpatient and outpatient services for individuals with TB, as well as better adherence to medication across all income groups [62] and among low-income individuals [63]
	Special health insurance packages [64, 65]			Increased access of economically disadvantaged individuals with TB to public health services [65] Reduction in catastrophic costs [64]
	Reimbursement of direct TB diagnostic costs [66]			Financial protection and enhanced detection of new TB cases [66]
Expanded rights	Allocation of a larger proportion of the GNP to social protection programs [77]	- Increase treatment adherence [70, 74–76, 78, 80] and treatment success rates [75, 80] - Assist with the costs associated with disease treatment [72, 80]	- Minimum age of 15 years [67], or adults ≥ 18 years in initial assessment for TB [71] - New cases diagnosed with confirmed pulmonary TB based on clinical criteria [67], with DR-TB who received integrated support for a minimum of three months [73], or with MDR-TB [80] - Absence of a history of MDR-TB [67] - Receipt of at least one social benefit during TB treatment [67] - Situation of social and health vulnerability [70, 74], or at high risk of treatment loss to follow-up for TB [76] - Monitoring by municipal public primary health care services [67]	Reduction in TB incidence and mortality rates [77]
	Direct monetary benefits: BFP, retirement, sickness benefits, pension, and other financial aids [67, 69, 70, 79]			Higher proportion of cure among individuals receiving government and non-government benefits or only direct benefits [67]
	Indirect benefits: basic food baskets, free public transportation, discounted electricity tariff, housing programs, food acquisition programs, nutritional support, among others [67, 69, 70, 79]			
	Expanded set of rights: food, income, and transportation [76]			Higher treatment success rate in the beneficiary groups [76] Lower incidence of death and loss to follow-up [73]

*BFP* Bolsa Família Program; *BMI* Body Mass Index; B*PC Benefício de Prestação Continuada* (Continuous Cash Benefit); *CRESIPT* The Community Randomized Evaluation of a Socio-economic Intervention to Prevent TB; *DOT* Directly Observed Treatment; *DR-TB* Drug-resistant tuberculosis; *GNP* Gross National Product; *MDR-TB* Multidrug-resistant tuberculosis; *RNTCP* Revised National Tuberculosis Control Program; *TB* Tuberculosis

#### Right to proper nutrition and nourishment

The right to proper nutrition and nourishment was established through specific measures and strategies for people affected by TB, characterized as governmental [20–24] and non-governmental [25–28] initiatives, and took place in two forms.

The first form involved the direct provision of monthly food vouchers [27, 29], weekly food baskets [28], or monetary values intended for the purchase of basic food baskets [20, 22, 25], with one of them being facilitated by the intervention of the World Food Programme (WFP) [20]. Additionally, food items were provided through the Afghanistan Food Assistance Program [24]. These measures were targeted at individuals with TB and low income who were undergoing directly observed treatment (DOT) [27] or conventional TB treatment [24], as well as the Australian Aboriginal population [28].

In Brazil, the provision of food vouchers improved TB treatment outcomes, with a 13% higher cure rate (RR = 1.13, 95% *CI:* 1.03–1.21) in the intervention group compared to the traditional treatment group [29], and a higher treatment completion rate (90.0% vs. 86.4%, *P* < 0.01) for those who received vouchers in Singapore [27]. The supply of basic food baskets also increased treatment success rates (88.0% vs. 60.5%, *P* = 0.001), reduced treatment failures (0.3% vs. 0.9%, *P* = 0.002), and decreased lost to follow-up rates (7.1% vs. 34.3%, *P* = 0.001) in Angola [20]. It also led to a significant increase in weight and body mass index (BMI) among individuals with active TB in India [25], as well as complemented TB treatment and provided immediate support to Aboriginal families affected by TB in Australia [28].

In the second form, this right was consolidated through nutritional support [21–23, 30], such as the monthly provision of rice and lentils to TB-affected people living below the poverty line [26], nutritional counseling, vitamin supplementation (vitamins A and B6), and fortified/therapeutic foods, especially for TB patients with varying degrees of malnutrition classified into four categories based on BMI [23].

Nutritional support contributed to the improvement of nutritional status and quality of life for people affected by TB in Ethiopia [21], although in some cases, the coverage and utilization of these measures were low and there were delays in receiving the benefits in India [22, 30]. Furthermore, they were associated with a 50% reduction in treatment failure (RR = 0.51, 95% *CI:* 0.30–0.86) in India [26], a decrease in the risk of lost to follow-up, with nutritional counseling leading to a 23% reduction and vitamins contributing to a 12% reduction in this risk in Kenya [23].

#### Right to income

The right to income was established through specific or sensitive strategies, or both, aimed at people affected by TB and were divided into two forms: income transfer programs and financial support, funded either by the government or non-governmental sources.

Specific government income transfer programs were implemented conditionally for individuals with sensitive TB [31–39] and those with antimicrobial-resistant TB [35, 39, 40], ranging in amounts from USD 15 [31] to USD 230 [38]. Some of these programs also included home visits, community meetings for health education, empowerment, reduction of TB-related stigmas [38], psychological support, and DOT [40].

Conditional income transfers by the Nigerian government in partnership with the Nigerian National TB Program demonstrated high acceptance levels, utilized by people with TB to purchase food, supplements, medications, transportation, and other additional personal needs. This also increased their enthusiasm and adherence to treatment [31]. In India, not receiving this benefit was associated with a five-fold higher likelihood of unfavorable treatment outcomes (95% *CI:* 2–12) [32], however, individuals with TB waited an average of 84 days (ranging from 45 to 120 days) to receive this benefit [33]. In China, for individuals with MDR-TB, financial support had a direct positive effect (b = 0.769,* P* < 0.001) and a positive indirect effect on treatment success, mediated by a self-reported social support scale (b = 0.541, *P* = 0.008; b = 0.538, *P* = 0.001) [40].

In Argentina, through Regulatory Decree 170/91 of Law 10,436, which established a legal framework to ensure socioeconomic protection for people affected by TB, successful TB treatment rates of 83% were evidenced, with a 11% loss to follow-up in the registered program group, compared to 69% success and 20% loss to follow-up in the non-registered group [34]. For individuals with MDR-TB who received the benefit, statistically significant differences were identified in the positive treatment outcome of 81.5% compared to 58.9% for those who did not receive the benefit [35].

In Peru, the project titled “The Community Randomized Evaluation of a Socio-economic Intervention to Prevent TB (CRESIPT)”, funded by both government and non-governmental sources, took place based on the conditions of individuals with TB, wherein if they had MDR-TB, the person would receive more money [36]. This project reduced the probability of incurring catastrophic costs (95% *CI:* 22–38%) for families of TB patients by 30%, compared to 42% (95% *CI:* 34–51%) for families that were not part of the project [37, 39]. Regardless of family poverty, successful TB treatment rates were 64% in the intervention group and 53% in the control group (*OR* = 1.6, 95% *CI:* 1.0–2.6) [38].

Regarding sensitive income transfer programs for people affected by TB, all studies analyzed the *Bolsa Família* Program (BFP) [41–50], and two studies examined the BFP in conjunction with the Continuous Cash Benefit (BPC—*Benefício de Prestação Continuada*), both funded by the Brazilian government [42, 43]. It was evidenced that the BFP led to a 7.6% higher cure rate and a 7% lower loss to follow-up proportion in the BFP beneficiary group compared to the non-BFP group [41]. Other studies found that being a BFP beneficiary improved cure rates by 8% (95% *CI:* 0.05–0.10) [43] and 5.2% [50], when compared to the group of individuals without income transfer benefits and TB.

Furthermore, people with TB enrolled in the BFP were also 7–11% more likely to achieve successful treatment outcomes than the control group [44]. In Brazilian municipalities with high BFP coverage, the TB incidence rate (*OR* = 0.96, 95% *CI:* 0.93–0.99) [46] and TB mortality rate (RR = 0.88, 95% *CI:* 0.79–0.97) [45] were significantly reduced compared to those with low and medium coverage. Among the indigenous population, it was identified that the BFP had a protective effect against active TB [48].

In terms of specific financial support, various studies have introduced monthly incentives offered to individuals affected by drug-sensitive TB [51, 52], MDR-TB [53], as well as to family members of individuals with TB [54], and households with individuals suffering from TB-related poverty [55]. These incentives varied in monthly amounts from USD 15 [52] to USD 200 [53]. Two studies mentioned funding for these measures from the Global Fund [54, 56], others from TB Control Programs [51, 52], and one study through a specific consortium for MDR-TB patients [53].

Financial incentives have resulted in improved treatment outcomes among individuals with MDR-TB, achieving an 82.4% cure rate [53]. Moreover, higher rates of treatment success (97.7%) [51], as well as rates of 92% [54] and 86% [52], were observed within the intervention groups, in contrast to 63% [54] and 71.1% [52] in the control groups. Notably, there was a significant decrease in loss to follow-up during the intervention period (20.2% vs. 5.0%, *P* < 0.001) [52].

While one study has indicated that financial support serves as an effective policy for MDR-TB control by expediting diagnosis and fostering treatment adherence [56], another study has highlighted that no single, specific, or TB-sensitive approach may be sufficient to mitigate the potentially catastrophic costs faced by individuals with antimicrobial-resistant TB [55].

#### Right to housing

The right to housing was identified in a study aimed at the homeless population, where a housing provision package was offered in the Republic of Korea, with support from the Ministry of Health and Welfare of the Republic of Korean government. This strategy, considered specific to TB, resulted in a treatment success rate of 96.1% (95% *CI:* 92.3–99.9). In addition to housing, this package also included meals at local restaurants, DOT, and case management by a healthcare team until the conclusion of TB treatment [57].

#### Right to health insurance

In countries with health systems operating under the logic of universal health coverage, such as China, the right to health insurance has been facilitated through sensitive strategies that include people affected by TB. Health insurance, in the context of universal health coverage, is a mechanism aimed at ensuring that all individuals have equitable access to necessary healthcare services, regardless of their ability to pay or financial situation.

One of these strategies involved reimbursements for TB services under the New Cooperative Medical Scheme, which was financed through a public–private partnership [58–60]. However, this reimbursement policy was inadequate for TB treatment [59] and did not exhibit a statistically significant difference (*P* > 0.05), indicating only a slight decline in the severity of catastrophic costs during TB treatment [58, 60].

Another non-governmental strategy, specifically designed to reimburse individuals with TB, was the China-Gates TB Project—Phase 2. This project identified a significant financial burden for individuals affected by TB, with over 82.5% of those living below the poverty line spending more than 10% of their family income on healthcare, and the majority allocating 40% of their non-food expenses to health services [61]. Nevertheless, other studies have demonstrated the effective improvement of this initiative in terms of access and utilization of TB services, both inpatient and outpatient, as well as better medication adherence across all income groups [62] and particularly among low-income individuals [63].

Conversely, two studies conducted in India provided special health insurance packages for individuals with MDR-TB in both the public and private sectors, linked to the Revised National Tuberculosis Control Program (RNTCP) [64, 65]. Consequently, this strategy increased the accessibility of healthcare services for poor people with TB [65] and reduced catastrophic costs by means of savings in health expenditures due to this innovative social protection mechanism [64].

Another study conducted in Bangladesh, through a collaboration between the TB Control Program and funding from the Global Fund, introduced the reimbursement of direct costs due to TB diagnoses as a means of financial protection. This approach aimed to connect the poorest people to diagnosis and improve the detection of new cases [66].

#### Expanded rights

Several studies have explored expanded rights aimed at providing social protection for people affected by TB through a series of measures and strategies that have enabled the realization of rights such as proper nutrition and nourishment, social assistance, housing, social security, income, and transportation [67–82]. In one of these studies, it was identified that countries allocating a higher proportion of their gross national product (GNP) to social protection programs are associated with a decrease of 8.16% and 5.48% in TB incidence and mortality rates, respectively [77].

Other studies have presented a broader, yet conditional, perspective on the right to social protection, encompassing both government and non-government programs and measures that are sensitive to TB. These programs include direct monetary benefits such as the BFP, retirement benefits, sickness allowances, pensions, and other forms of financial aid. Additionally, there are indirect benefits like food baskets, free public transportation, discounts on electricity tariffs, housing initiatives, food acquisition programs, nutritional support, among others [67, 69, 70, 79].

These studies have revealed a higher rate of cure among individuals receiving both government and non-government benefits (90.5%) or solely direct benefits (81.6%) [67]. Furthermore, in three qualitative studies, although the absence of specific initiatives for people with TB was identified in Brazil based on statements from healthcare service managers [79], in countries such as India and South Africa, people with TB perceive these initiatives as crucial strategies for promoting treatment adherence [70]. This is noteworthy even in the face of the substantial burden that TB places on their lives and the lives of their families, coupled with limited access to social protection [69].

Conversely, studies that have proposed strategies specifically tailored to TB-related social protection indicate that when these strategies are provided through an expanded range of measures encompassing rights to proper nutrition, income, and transportation, the success rate of treatment is significantly higher among beneficiary groups [76]. Moreover, there is a lower incidence of mortality (*OR* = 0.876, 95% *CI:* 0.811–0.947;* P* = 0.0009) and loss to follow-up (*OR* = 0.752, 95% *CI:* 0.597–0.873; *P* = 0.0023) [73].

Furthermore, people with TB have expressed that cash incentives, food provisions, or transportation vouchers have facilitated their return to healthcare services, thereby ensuring continuity of treatment [71]. For those afflicted with MDR-TB, the necessity for incentives has been found to surpass what is provided by the TB Control Program [82]. From the perspective of healthcare professionals and managers, these incentives are pivotal for fostering treatment adherence among individuals with TB, particularly those with limited purchasing power and who find themselves in socially vulnerable situations [74]. Four studies have highlighted discrepancies in terms of equity of access to such social protection initiatives, considering aspects like approach, availability, resources, accessibility, and suitability [72, 75, 78, 81].

## Discussion

The stagnation in the global decline of TB incidence has prompted several countries to incorporate health programs and social policies into the realm of healthcare provision. This stands in contrast to the traditional curative and biomedical approach, all in pursuit of the End TB Strategy’s goal to eliminate TB by 2050 [44, 77]. It is widely acknowledged that TB is a consequence of social inequalities. Despite certain measures implemented throughout the course of TB treatment to alleviate the affliction experienced by those affected [74], these efforts fall short of achieving the intended goal.

In light of this, social protection emerges as a robust policy within the context of TB and a fundamental pillar of societal structure. It offers safeguards to individuals and families against poverty, social exclusion, challenges in workforce integration, income inadequacy, and food insecurity. Consequently, it contributes significantly to addressing the disease [44, 69, 74, 77]. Moreover, in countries with healthcare systems built upon the principle of universal coverage, social protection plays a pivotal role in enhancing access to affordable healthcare services [77].

A study has brought to light that countries channeling a substantial portion of their GNP into social protection witness lower rates of TB prevalence, incidence, and mortality [77]. While the primary responsibility for social protection programs or policies rests with government administrative bodies, the integration between TB control programs and both governmental and non-governmental institutions prove to be indispensable. This integration aims not only at reducing the rate of loss to follow-up, boosting adherence and therapeutic success, but, more crucially, at mitigating the social, economic, and programmatic burdens imposed by TB [20, 21, 24, 29, 77].

This is because when a social protection program has a governmental foundation, strong political commitment, and financial resources, it becomes possible to envision achieving the global target of 85% success in TB treatment [53, 54]. Therefore, without access to effective public resources, the access to treatment, completion, and cure of TB is compromised, which can lead to disease aggravation, and even death. This situation also results in high transmission rates and higher costs for health and social care services [72].

Regarding the right to proper nutrition and nourishment, it is identified that deprivation of the same contributes to the illness and worsening of TB [24]. Thus, the success of strategies based on food vouchers, food baskets, or nutritional support in treating the disease reveals aspects of inclusion and human rights for people affected by TB in social development policies. In addition, these strategies serve as support for families and households facing food insecurity and struggling with eating habits that lead to malnutrition [23–25, 29, 69, 74].

It is understood that the incidence of TB is disproportionately high among people living in poverty [43, 69]. Due to this, specific or sensitive economic protection schemes for TB is endorsed to prevent catastrophic costs. These schemes have proven essential in helping reduce the stigma associated with TB illness [52]. Moreover, they play a crucial role in motivating individuals affected by the disease to seek treatment [71]. This, in turn, improves adherence and treatment outcomes in areas with high TB incidence [32, 34, 35, 37, 38, 43, 45, 46, 50, 51, 55].

One of the most significant examples of this is the Brazilian government’s BFP, which forms part of comprehensive social protection policies and serves as a sensitive strategy to combat inequalities while mitigating the adverse effects of poverty [41, 43, 45, 48–50]. This conditional cash transfer program has increased the likelihood of TB cure, particularly by addressing healthcare-seeking behaviors, improving treatment response, ensuring food security, and enhancing nutritional status among beneficiaries [50]. However, it has been identified that this effect could be further amplified if the program specifically included people affected by TB, extending beyond low socio-economic level families [41].

The development, implementation, effectiveness, and expansion of programs and strategies aimed at providing economic support are not only crucial for national and global TB response policies [36, 39, 42], but they also bolster social protection policies. These initiatives bring about significant improvements in the living conditions of the population by enhancing access to formal education and promoting high-quality dietary standards. Additionally, they facilitate greater income generation and provide access to health services and the labor market [42, 45, 48].

Nevertheless, a study has identified that no specific or TB-sensitive economic approach alone can be sufficient to avert the catastrophic costs resulting from the disease [55]. This highlights the need for investments in the universal implementation of social and health policies. Above all, there is a pressing requirement for social protection to be extended to individuals and families affected by TB [45, 55]. Furthermore, comprehensive evaluations on a large scale are imperative to assess the effect of socioeconomic support on TB treatment, care, and prevention. These assessments play a pivotal role in addressing aspects that can significantly contribute to the elimination of this prominent public health issue [38].

In this regard, certain refinements are recommended in the policies concerning economic protection for people with TB. These refinements encompass increasing the frequency and amounts of conditional income transfers, covering individual members of families affected by the disease. The aim is to enhance adherence to preventive TB therapy while also bolstering social support, particularly for individuals at a higher risk of loss to follow-up, such as those experiencing homelessness, migrants, individuals with some form of drug or alcohol dependency, and those with drug-resistant TB [39, 72, 76].

Housing protection, ensured through the right to housing, has yielded positive effects on treatment outcomes for individuals facing homelessness and TB. By creating a welcoming environment, it has mitigated the stigma and discrimination often associated with hospitalizations in medical facilities due to social reasons [57]. Furthermore, given the multifaceted and diverse vulnerabilities of individuals experiencing homelessness, a person-centered approach to care and integration into the national social assistance policy have guaranteed the inclusion of these individuals in state-provided social protection for a minimum of two years following the completion of TB treatment [57].

In countries where universal state-funded healthcare systems are absent, TB places a significant financial burden on families affected by the disease. This underscores the need to formulate policies that concentrate on safeguarding against financial risks, exemplified by tailored health insurance schemes for TB [60]. In this context, studies have identified the effectiveness of linking national health insurance with the national TB control programs. This linkage is achieved through the provision of specialized packages catering to individuals with both sensitive TB and MDR-TB. This approach has notably contributed to reducing the time gap between diagnosis and the initiation of treatment [64, 66].

On the other hand, the reduction in reimbursement from a Chinese health insurance program for individuals with TB has resulted in substantial financial burdens and has not provided complete protection, particularly for those with lower incomes, who have incurred catastrophic health costs [58, 60, 61, 63]. Therefore, reimbursement policies should be improved through appropriate institutional arrangements, linking and coordination with national TB control programs, aiming to enhance financial protection by including more comprehensive healthcare coverage, along with other more effective interventions to alleviate the financial burden of TB and reduce unnecessary hospitalizations for these individuals [58–61, 65].

It is important to highlight that, although measures and strategies for the social protection of people affected by TB have shown a significant effect on addressing the disease, some barriers to access and implementation still persist. Examples include the lack of provision of social incentives or associated costs to access them [69, 75, 79, 80]. Moreover, issues such as the availability and validation of a bank account, the complexity of the process, and the requirement of documents and conditionalities to receive benefits, combined with the social stigma of TB, the cultural norms of the population, operational errors, and internal micropolitics of states, disproportionately affect the most vulnerable individuals [22, 33, 49, 69, 81].

To overcome these challenges, synergy between social protection interventions and national TB control programs can prove to be effective [34]. For this purpose, policymakers should prioritize administrative aspects and strengthen intersectoral collaboration between the health and social assistance areas. This can be achieved through the joint creation of protocols, training, and education of healthcare professionals, aimed at recognizing the needs of people with TB and raising awareness about existing incentives as rights. Additionally, emphasis should be placed on understanding the duration, values, and conditions that must be met for their receipt. By taking these steps, the expansion, operationalization, and implementation of measures and strategies aimed at social protection can become a central pillar of prevention and care activities for people affected by TB [30, 33, 71, 79].

## Research gaps

This scoping review identified primarily specific measures and strategies aimed at the social protection of people affected by TB, especially in countries with a high disease burden. These measures, viewed from a rights perspective, involved conditions for access and temporarily alleviated the challenges faced during TB treatment. However, since these strategies are more targeted, they lack the ability to permanently alter the structural conditions of society, such as poverty, illiteracy, and other social determinants of health that are intrinsic to TB illness and the ongoing transmission chain.

In this regard, further research should be conducted to evaluate the effect of sensitive interventions proposed or implemented to address social inequalities and health inequities that impact TB. The goal is to break the cycles of poverty, destitution, and sustainable income generation. For example, even though no study presented measures and strategies focused on employment market protection, authors recommend the incorporation of people with TB into social and labor safety networks, as well as income-generating activities that include job training [21, 79]. Thus, in line with the objectives of The End TB and the SDGs, discrepancies related to the right to proper nutrition and nourishment, income, housing, health insurance, and other rights encompassing TB management must be addressed and overcome.

## Limitations

This study aimed to broadly explore the specific contextual mechanisms of measures and strategies for the social protection of people affected by TB as a right. However, some interventions or practical experiences may not have been included because they were published before 2015 or in languages other than English, Portuguese, or Spanish. Furthermore, we chose to exclude gray literature because we understand its nature to be publications aimed at providing guidance on measures to be adopted or strategies to be implemented in the context of social protection and TB management.

Another challenge in this review is that many of the interventions and programs described are not unimodal, meaning that several interventions are integrated into a single strategy, making it difficult to identify which component caused the change in outcomes.

The results presented by this review in the conceptual framework should be interpreted with caution due to the different epidemiological and socioeconomic scenarios in which the measures and strategies were implemented, the organizational and operational aspects, as well as the format of the healthcare system implemented in different countries.

It is also worth noting as a limitation of this study the lack of assessment of the quality of scientific evidence found and the absence of formal consultation with stakeholders on the subject in question.

## Conclusions

This scoping review systematically synthesized the scientific evidence addressing measures and strategies related to social protection of people affected by TB, which encompassed the right to proper nutrition and nourishment, income, housing, and health insurance, as well as expanded rights involving social assistance and social security. It was identified that ensuring such rights contributes to the improvement of nutritional status and quality of life for people with TB, also as the reduction of catastrophic costs, increased access to health measures and services, and adherence to treatment, leading to higher rates of favorable treatment outcomes. The conceptual framework of this study aided in understanding organizational and operational aspects, as well as in the planning, execution, and potential effects of social protection measures and strategies in addressing TB.

The findings of this study can be useful in guiding the development of evidence-based social and health policies and joint decision-making between TB control programs and governance structures that encompass the right to proper nutrition and nourishment, income, housing, and health insurance, in addition to other expanded rights encompassing social assistance and social security.

### Supplementary Information

**Additional file 1.** Search strategies for a scoping review on social protection as a right of people affected by tuberculosis.

## Data Availability

Data sharing is not applicable to this article as no datasets were generated or analysed during the current study.

## Abbreviations

BFP: Bolsa Família Program
BMI: Body Mass Index
*BPC*: *Benefício de Prestação Continuada* (Continuous Cash Benefit)
CINAHL: Cumulative Index to Nursing and Allied Health Literature
CRESIPT: The Community Randomized Evaluation of a Socio-economic Intervention to Prevent TB
*DeCS*: *Descritores em Ciências da Saúde* (Health Sciences Descriptors)
DOT: Directly observed treatment
DR-TB: Drug-resistant tuberculosis
GNP: Gross National Product
LILACS: Latin American and Caribbean Health Sciences Literature
MDR-TB: Multidrug-resistant tuberculosis
MEDLINE: Medical Literature Analysis and Retrieval System Online
MeSH: Medical Subject Headings
PRISMA-ScR: Preferred Reporting Items for Systematic Reviews and Meta-Analyses Extension for Scoping Reviews
RNTCP: Revised National Tuberculosis Control Program
SDGs: Sustainable Development Goals
TB: Tuberculosis
WFP: World Food Programme
WHO: World Health Organization
